# Real-Life Management of Diabetic Macular Edema with Dexamethasone Intravitreal Implant: A Retrospective Analysis of Long-Term Clinical Outcomes

**DOI:** 10.1155/2020/4860743

**Published:** 2020-04-10

**Authors:** Massimo Nicolò, Donatella Musetti, Maria Marenco, Lorenzo Cotti, Monica Bonetto, Mauro Giacomini, Carlo Enrico Traverso

**Affiliations:** ^1^Clinica Oculistica, DiNOGMI, Università di Genova, Ospedale Policlinico San Martino IRCCS, Genova, Italy; ^2^Fondazione per La Macula Onlus, Genova, Italy; ^3^Healthropy Srl, Savona, Italy; ^4^DIBRIS, University of Genova, Genova, Italy

## Abstract

**Purpose:**

Inflammation plays a key role in the pathogenesis of diabetic macular edema (DME), and intravitreal corticosteroids are among the recommended therapies. The goal of this retrospective analysis was to describe outcomes with dexamethasone intravitreal implant (DEX implant) in real life.

**Methods:**

Medical digital records of DME patients treated with DEX implant and followed up for 3 years were analyzed. Treatment with DEX implant was started either as first-line therapy in pseudophakic patients and in patients with cardiovascular comorbidities or as second-line therapy in patients refractory to the inhibitor of the vascular endothelial growth factor (anti-VEGF) therapy. Analyzed outcomes included central macular thickness (CMT) and best-corrected visual acuity (BCVA). Mean number of implant injections per patient and mean duration of the interval between injections were also estimated.

**Results:**

Seventy-five patients (mean age 65.7 (±12.3) years; 53 phakic and 22 pseudophakic) with DME were included. Overall, 84 eyes were treated. Mean CMT improved from 380.1 (±100.3) *µ*m at baseline to 306.8 (±77.0) *µ*m at 36 months (*p*=0.0003). Mean BCVA improved for up to 6 months (*p*=0.08) and then started to decrease reaching values lower than baseline after 24 months. In pseudophakic patients, BCVA improvements were more pronounced and sustained up to 36 months (*p*=0.6). Over 36 months, each patient received on average 2.4 (±1.6) intravitreal injections of DEX implant. The time interval between consecutive injections was included between 180 and 240 days. No unexpected safety issues were reported.

**Conclusions:**

With fewer than 3 injections per patient over a 3-year period, DEX implant was able to improve anatomic outcomes in DME patients. Only pseudophakic eyes showed also a long lasting functional benefit at 36 months.

## 1. Introduction

Diabetic macular edema (DME) is characterized by the accumulation of fluid within the central portion of the retina and by macular thickening caused by blood-retinal barrier dysfunction [1]. DME, which results in reduced visual acuity and is a major cause of visual loss, is estimated to affect approximately one-fifth of patients with diabetic retinopathy [1–3]. Inflammation, with leukostasis on the surface of retinal capillaries as one of the earliest events, is crucially implicated in the breakdown of the blood-retinal barrier that leads to increased permeability and the development of DME [4, 5].

Standard care for DME has long been based on diabetes medical control and laser photocoagulation therapy. In 2012, DME management changed dramatically due to the introduction of the first pharmacologic therapy for this condition, ranibizumab, an inhibitor of the vascular endothelial growth factor (VEGF) [6, 7]. Anti-VEGF agents have since rapidly emerged as first-line therapy for DME [1]. However, evidence from trials, as well as from real-life observational studies, has shown that significant proportions of DME patients have an incomplete response or fail to respond to anti-VEGF therapy [1, 8]. In addition, anti-VEGF agents are administered by intravitreal injection on a monthly basis, which may represent a considerable burden for many patients. Furthermore, anti-VEGF agents are not indicated for DME patients with a history of major cardiovascular events [1]. Therefore, there is a need for additional approaches to the treatment of DME [3].

Intravitreal corticosteroids may be a valid approach to DME treatment as they produce an anti-inflammatory effect via various mechanisms, including decrease in the synthesis of inflammatory mediators and VEGF, inhibition of leukostasis, and enhancement of the barrier function of vascular endothelial cell tight junctions [1, 3]. Intravitreal corticosteroids may, therefore, exert a more comprehensive effect on the inflammatory cascade than anti-VEGF agents [1]. Currently, intravitreal corticosteroids are an important component in the armamentarium of drugs for DME, but they are mostly used as the second-line therapy, after failure of anti-VEGF therapy. According to the guidelines issued in 2017 by the European Society of Retina Specialists (EURETINA), in nonresponders who have already been treated with 3 to 6 anti-VEGF injections, it is reasonable to switch to a corticosteroid [1].

Dexamethasone intravitreal implant (DEX implant, Ozurdex®, Allergan, Inc, Irvine, CA, USA) is a sustained-release corticosteroid developed to reduce the need for frequent intraocular injections [9]. The biodegradable implant releases dexamethasone into the vitreous over a period of ≤6 months [9]. The efficacy and safety of DEX implant have been investigated in randomized trials [3, 10–12], in head-to-head comparisons with anti-VEGF agents [13, 14], and in a number of real-life studies [15–19]. In the MEAD trial, for example, DEX implant 0.7 mg or 0.35 mg provided substantial long-term improvements in visual acuity and central macular thickness (CMT) in patients with DME, with a mean of only 4 to 5 injections over 3 years [3]. The proportion of patients with a ≥15-letter gain in best-corrected visual acuity (BCVA) at the end of the study was significantly greater with DEX implant compared with sham injection. Repeated DEX implant injections were associated with increased development or progression of cataract in phakic eyes (i.e., eyes with their natural lens) and in elevations of intraocular pressure (IOP). Both cataract progression and IOP increases are expected and manageable complications of corticosteroid treatment. The small number of intravitreal injections needed with dexamethasone implants may represent a significant reduction in the treatment burden for patients [3].

DEX implant 0.7 mg was approved in 2014 by the European Medicines Agency (EMA) for the treatment of adult patients with visual impairment due to DME who are pseudophakic (i.e., have undergone cataract surgery), are insufficiently responsive to, or unsuitable for noncorticosteroid therapy [9]. According to the EMA label, retreatment may be performed after approximately 6 months if the patient experiences decreased vision and/or an increase in retinal thickness; concurrent administration in both eyes is not recommended [9]. Ways to optimize treatment with DEX implant are under investigation; evidence from real-life observations and exploratory studies suggests that personalized, as-needed regimens with flexible injection intervals and close monitoring may be effective in ensuring vision and anatomic benefits [12, 15, 17, 18].

We report here the results of a retrospective analysis of the medical digital records of DME patients treated with DEX implant and followed up for 3 years at an ophthalmologic clinic in Italy. The aim of the study was to describe the pattern of DME treatment with sustained-release corticosteroids in routine clinical practice with a focus on long-term outcomes, retreatment intervals, and frequency of intravitreal injections.

## 2. Methods

### 2.1. Study Design and Patients

This was a retrospective analysis performed to describe patterns and outcomes of DME treatment with DEX implant in a setting of routine clinical practice. The study adhered to the tenets of the Declaration of Helsinki and was approved by the institutional review board. Informed consent was obtained from all patients.

Data from type 2 diabetic patients with diabetic macular edema and nonproliferative diabetic retinopathy (NPDR), treated with dexamethasone 0.7 mg intravitreal implant (Ozurdex®, Allergan, Inc., Irvine CA, USA) between June 2015 and June 2018 at an ophthalmologic clinic in Italy (Clinica Oculistica, DiNOGMI, University of Genova, Genova), were retrieved from Imaculaweb, a web-based platform designed to collect clinical information from routine ophthalmologic practice [20]. Both naïve and unresponsive to anti-VEGF therapy patients were included in the study. Patients were considered unresponsive to anti-VEGF treatment when macular edema with retinal thickness of >250 *µ*m in the central subfield as detected by SD-OCT (DRI 3D OCT-2000 or Swept Source DRI OCT Triton, Topcon) did not improve after at least three consecutive anti-VEGF (ranibizumab or aflibercept) injections applied once a month. Exclusion criteria were age ≤18 years and a positive history of glaucoma and/or elevated IOP. Following the administration of DEX implant, all patients were monitored for up to 36 months. During the follow-up period, no patient underwent laser treatment. The following data were collected from the I-macula web platform: demographic and clinical characteristics of patients at baseline (treatment initiation), baseline ophthalmologic findings, optical coherence tomography (OCT) scans, and retinal fluorescein angiography, as well as ophthalmologic findings and OCT scans over 36 months of treatment with DEX implant. The number of implant injections received and the duration of the interval between consecutive injections were also collected.

### 2.2. Treatment

Sustained-release DEX implant was administered via pars plana intravitreal injection, following the instillation of anesthetic drops (oxybuprocaine hydrochloride, 4 mg/mL). In patients treated bilaterally, the second eye was treated 15 days after the first eye. DEX implant was administered either as the second-line therapy in patients previously treated with and unresponsive to anti-VEGF therapy or as the first-line therapy in patients with cardiovascular comorbidities, and therefore not eligible for anti-VEGF therapy, as well as in patients with pseudophakic eyes. Over the 36 months of follow-up, the administration of dexamethasone was repeated if needed, based on the presence of macular edema and/or subretinal fluid and a retinal thickness of >250 *µ*m in the central subfield as detected by SD-OCT (DRI 3D OCT-2000 or Swept Source DRI OCT Triton, Topcon.

### 2.3. Assessments

Before initiating the treatment with DEX implant, patients underwent a comprehensive eye examination and retina imaging using spectral-domain OCT (DRI 3D OCT-2000, Topcon) or swept-source OCT (Swept Source DRI OCT Triton, Topcon) and fluorescein angiography. Following the administration of the dexamethasone implant, patients were seen regularly with visits scheduled at 3, 6, 12, 18, 24, and 36 months from treatment initiation. At each control visit, patients underwent a complete eye examination, and an OCT scan was performed. Efficacy measures included BCVA assessed according to the Early Treatment Diabetic Retinopathy Study (ETDRS) method [21] and CMT measured on OCT images.

### 2.4. Statistics

Data were analyzed by descriptive statistics, including means and standard deviation for continuous variables and frequencies and relative frequencies (percentages) for categorical variables. Statistically significant data were calculated using the ANOVA for repeated measurements with a post hoc analysis test. Data were analyzed globally as well as after dividing the study population according to lens status (i.e., phakic and pseudophakic eyes).

## 3. Results

This retrospective analysis included 75 patients and a total of 84 eyes treated with DEX implant. Baseline patient characteristics are summarized in Table 1. Patients had a mean age of 65 (±12.3) years (range 53–81 years) and were predominantly male (61.3%). At baseline, mean CMT was 380.1 (±100.3) *µ*m, and mean BCVA was 55.0 (±18.4) letters. Twenty-two patients (29.3%) were pseudophakic, while the remaining 53 (70.7%) were phakic and did not undergo cataract surgery during the entire observation period. Naïve eyes were 45 (53.5%).

**Figure 1:**
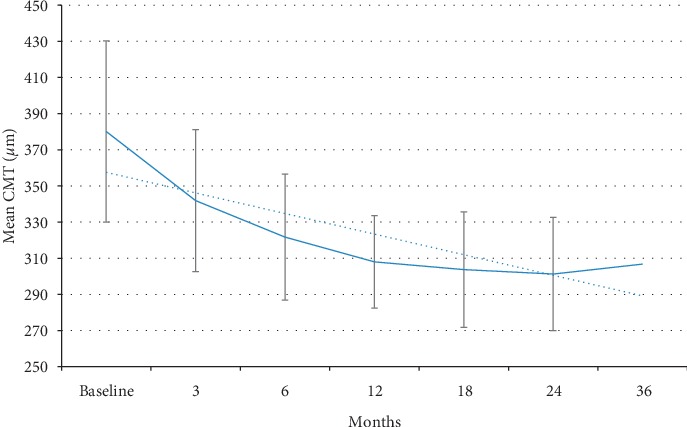
Changes in mean central macular thickness during treatment with sustained-release dexamethasone implant.

**Figure 2:**
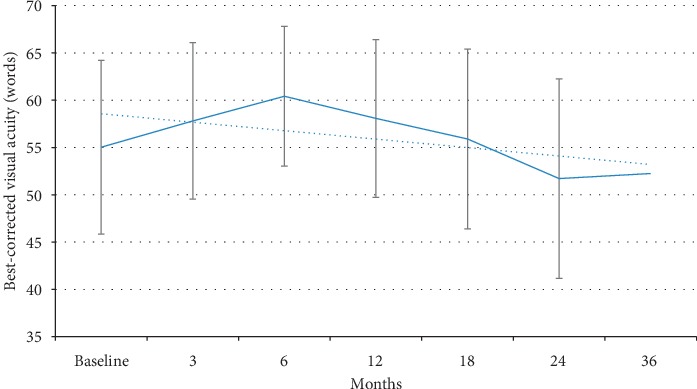
Changes in mean visual acuity during treatment with sustained-release dexamethasone intravitreal implant.

**Figure 3:**
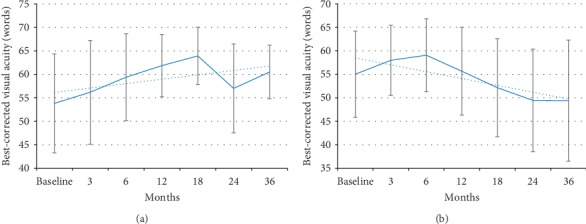
Changes in mean visual acuity during treatment with sustained-release dexamethasone intravitreal implant in pseudophakic (a) and phakic (b) patients.

Treatment with DEX implant was associated with a progressive and statistically significant decrease in mean CMT, from 380.1 (±100.3) *µ*m at baseline to 307.0 (±51.2) *µ*m at 12 months, 301.3 (±62.7) *µ*m at 24 months, and 306.8 (±77.0) *µ*m at 36 months (Figure 1). The decrease in CMT was already significant as early as the third month (*p*=0.03). This improvement was not paralleled by an increase in visual acuity in the overall population, which showed a trend, although not statistically significant, towards improvement during the first 6 months of treatment (gain of approximately 5 letters in BCVA) and then started to decrease reaching values lower than baseline at 24 and 36 months (Figure 2). The mean visual acuity at baseline was 55.02 ± 18.3 (SD) ETDRS letters. Maximal visual acuity was achieved by 6 months after initiating treatment (59.06 ± 14.9). By 3 years, the mean visual acuity fell below the baseline (49.4 ± 25.7 letters at year 3). Analysis of visual acuity according to lens status showed that in pseudophakic patients, mean BCVA progressively improved with treatment up to 18 months (gain of approximately 10 letters in BCVA) and remained better than the baseline value also at 24 (gain of 3 letters vs baseline) and 36 months (gain of 6 letters vs baseline) (Figure 3(a)). By contrast, in phakic patients, mean BCVA showed a slight improvement (<5 letters) during the first 12 months of treatment and then progressively decreased (Figure 3(b)). The presence and progression of cataract might have impacted visual acuity results.

**Figure 4:**
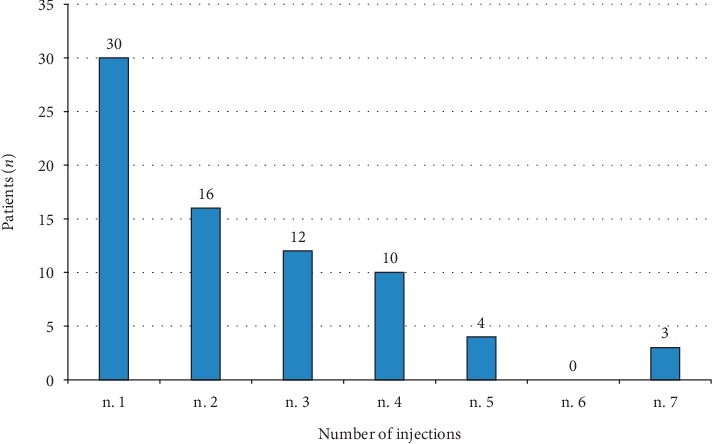
Number of dexamethasone implant injections per patient over 36 months of observation.

**Figure 5:**
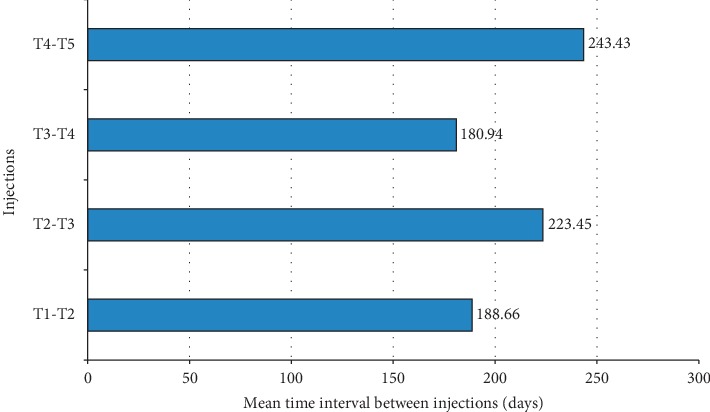
Mean interval duration between consecutive dexamethasone implant injections.

Thirty patients (40%) received a single implant of dexamethasone over 36 months, while the majority needed repeated treatments (Figure 4). In detail, 16 patients (21.3%) required 2 implants, 12 patients (16%) required 3 implants, 10 patients (13.3%) required 4 implants, and 7 patients (9.3%) received >4 implants over 3 years. The mean number of injections over 36 months was 2.4 (±1.6). The duration of the interval between two consecutive injections ranged from 180 days to 240 days (Figure 5). The proportion of eyes that required 3 or more injections during follow-up was 14 (31.1%) and 19 (48.7%) eyes in the naïve and previously treated group, respectively.

Intravitreal dexamethasone was overall well tolerated: no adverse events were reported following implant injection, and no unexpected safety issues were recorded throughout the observation period.

## 4. Discussion

According to this retrospective analysis involving 75 patients with DME, treatment with dexamethasone 0.7 mg intravitreal implant was associated with a substantial improvement in CMT (mean decrease >70 *µ*m from baseline to 3 years), in line with the findings from clinical trials and real-life observational studies [3, 16, 19]. Overall, the mean improvement in BCVA during the first 6 months of follow-up was of 5 ETDRS letters. This improvement was, however, not maintained during the following visits, most likely due to cataract progression associated with repeated corticosteroid treatment, as widely reported in the literature [3, 15, 22]. In support of this point is the observation that the subgroup of pseudophakic patients, who had already undergone cataract removal, achieved better visual outcomes than phakic patients, with a mean BCVA improvement of approximately 10 letters by 18 months. A notable finding of this retrospective analysis was the relatively low number of implant injections per patient: approximately 60% of the study population received over three years ≤2 implant injections and the mean number of implant injections per patient over the same period amounted to 2.4. The subgroup treated with ≤2 implant injections over three years included patients who had already received several cycles of anti-VEGF therapy with poor response. In some of these patients (40% of the entire study population), a single intravitreal injection of DEX implant was sufficient to achieve anatomic improvement, with no need for further injections. The mean time between consecutive intravitreal injections ranged from 6 to 8 months.

A few practical issues concerning the treatment of DME with DEX implant are still poorly defined, including the optimal interval between intravitreal injections. A number of studies have investigated retreatment time intervals and injection rates in the setting of real life [16–19]. Overall, these studies have reported higher injections frequencies compared to those highlighted by our analysis. De Geronimo et al. [23] recently reported a mean of 5.9 DEX implant during a mean follow-up period of 37.6 months. The RELDEX real-life study found that a mean of 3.6 injections was administered per patient over a 3-year follow-up, with a mean time to retreatment of 7.3 months [16]. A French survey on the use of DEX implant in clinical practice found that the average annual number of injections per patient was 2.4, and the average interval between treatments was 4.9 months [17]. According to a systematic review of real-life studies, the mean retreatment time was 5.3 months, and the mean number of injections was 1.3 every six months [18].

Outcomes in phakic versus pseudophakic patients have also been addressed by a number of studies [3, 15, 22]. In the MEAD trial, mean changes in BCVA from baseline were confounded by cataract in phakic patients after the first year of treatment with DEX implant [3]. In pseudophakic patients, visual outcomes were favorable and consistent over 3 years. In phakic patients who developed cataract, vision improved again after cataract extraction. Overall and as shown by our analysis, treatment of DME with intravitreal corticosteroids appears to be more effective in pseudophakic patients in terms of BCVA improvements [15, 24]. According to the EURETINA guidelines 2017, pseudophakic patients are preferred candidates for the use of steroids, while phakic patients should be informed about the high risk of cataract surgery [1].

Another debated issue concerning the treatment of DME with intravitreal corticosteroids is the timing of treatment initiation and the appropriateness of current treatment standards, which generally limit corticosteroid therapy to the second-line treatment [1, 23, 25]. A recent study investigated patterns of recurrence following a first intravitreal injection of DEX implant in patients with DME [26]. The study considered three patterns of recurrence: qualitative anatomic recurrence, quantitative anatomic recurrence, and functional recurrence. Qualitative anatomic recurrence was found to appear first, followed by anatomic quantitative recurrence and functional recurrence, suggesting that it may be an early sign of disease progression to functional loss. Therefore, early treatment to prevent qualitative anatomic recurrence may prevent loss of visual acuity.

With regard to the issue of whether to use DEX implant for the first- or second-line therapy, evidence from studies that have compared naïve and refractory DME patients suggests that naïve patients have greater benefits from intravitreal corticosteroids than patients refractory to previous pharmacologic therapies [8, 16, 27]. A retrospective study involving 79 eyes from 62 patients found that patients naïve to treatment for DME responded better to intravitreal dexamethasone as highlighted by a significantly longer treatment-free interval compared with those already exposed and refractory to anti-VEGF therapy (10.5 months versus 7.8 months, *p*=0.016) [27]. We found that the proportion of eyes requiring more than 3 injections during follow-up was lower in naïve versus previously treated patients (31.1% versus 48.7%). Improvements in visual acuity and decreases in CMT were reported in both groups. These observations are in accordance with de Geronimo et al [23] who demonstrated that a numerically smaller DEX-I injections are required in patients naïve. This may be explained by the better anatomical integrity of the retina in naïve patients compared to those with other agents. The real-life study RELDEX also found that treatment-naïve patients had substantially longer times to retreatment with DEX implant than patients who had proven refractory to anti-VEGF therapy [16].

The present retrospective analysis also confirms the safety profile of DEX implant that emerged from the clinical trials and from real-life studies in DME patients [3, 28, 29]. Patients, including those who received multiple injections and bilateral injections, tolerated the treatment well.

Besides the small sample size, this study has the limitations typically associated with a retrospective design, such as lack of randomization, missing data, and patients lost to follow-up. Moreover, the lack of data at month 1 and 2 may have led to miss some IOP spikes. Furthermore, it was not well known yet that the retreatment interval in real life should be shorter than 6 months as several publications in the literature have demonstrated so far. This study provides, however, additional information about the real-life use of DEX implant for the treatment of DME and highlights the potential of this approach in patients refractory to anti-VEGF therapy.

## 5. Conclusions

Treatment of DME patients, in a setting of routine clinical practice, with an average of 2.4 injections of DEX implant was associated with a substantial decrease in CMT over a 3-year period. In the subgroup of pseudophakic patients, this treatment was also associated with progressive improvements in visual acuity from baseline to 18 months. DEX implant was well tolerated, with no unexpected safety issues reported over the 3 years of observation. Some features of DME treatment with DEX implant, including treatment initiation and retreatment criteria, need to be better defined. An additional research effort is required also for identifying DME patients who may benefit from early, first-line treatment with intravitreal corticosteroids.

## Figures and Tables

**Table 1:** Demographic and clinical characteristics at baseline.

	Overall *n* = 75	Phakic patients *n* = 53	Pseudophakic patients *n* = 22
Gender, *n* (%)			
Male	46 (61.3)	33 (62.3)	13 (59.1)
Female	29 (38.7)	20 (37.7)	9 (40.9)
Age, yr	65.7 (±12.3)	66.2 (±9.7)	66.1 (±9.9)
CMT, *µ*m	380.1 (±100.3)	385.4 (±110.6)	369.0 (±76.6)
Visual acuity, ETDRS letters	55.0 (±18.4)	55.0 (±18.4)	53.8 (±21.1)

Unless otherwise stated, data are presented as mean values (±standard deviation). CMT: central macular thickness; ETDRS: Early Treatment Diabetic Retinopathy Study.

## Data Availability

All the data used to support the findings of this study are available from the corresponding author upon request.
